# Preliminary antifibrotic and vasoconstrictor effects of adrenaline in Schlemm’s canal and suprachoroidal minimally invasive glaucoma surgery in primary open-angle glaucoma

**DOI:** 10.1007/s00417-024-06642-3

**Published:** 2024-09-30

**Authors:** Jinyuan Luo, Julia Fajardo-Sanchez, Mengqi Qin, Brihitejas Patel, Karishma Mahtani, Henrietta Ho, Cynthia Yu-Wai-Man

**Affiliations:** 1 Faculty of Life Sciences & Medicine, King’s College London, London, SE1 7EH UK; 2 Department of Ophthalmology, Guy’s and St Thomas’, NHS Foundation Trust, London, SE1 7EH UK; 3 Department of Ophthalmology, Renmin Hospital of Wuhan University, Wuhan, 430060 China

**Keywords:** Glaucoma, Adrenaline, Fibrosis, MIGS

## Abstract

**Purpose:**

To investigate the antifibrotic and vasoconstrictor effects of adrenaline in Schlemm’s canal and suprachoroidal minimally invasive glaucoma surgery (MIGS).

**Methods:**

Human trabecular meshwork (TM) cells were treated with different concentrations of adrenaline (0%, 0.0005%, 0.01%), and we measured the effects on contractility, cell viability and the expression of key cell cycle and fibrosis genes. Adrenaline 0.05% was also injected intracamerally in five primary open-angle glaucoma patients undergoing iStent inject or MINIject surgery combined with phacoemulsification. All patients were assessed for ocular and systemic adverse reactions, including the effects on intraoperative pupil size, preoperative and postoperative visual acuity, intraocular pressure, and anterior segment OCT results.

**Results:**

Adrenaline significantly reduced the contractility of TM cells in a dose-dependent manner (87.8%, 80.6%, 7.9% matrix contraction with adrenaline 0%, 0.0005%, 0.01%, respectively). Adrenaline did not exhibit any significant cytotoxicity even at high concentrations (*P* > 0.05). Adrenaline 0.01% significantly downregulated the expression of key cell cycle genes in the G2 and M phases, and also decreased the expression of *MRTFB* and *ACTA2* genes (*P* < 0.05). Intracameral injections of adrenaline 0.05% in the five MIGS patients did not result in any ocular or systemic adverse effects.

**Conclusion:**

We recommend intracameral injections of adrenaline 0.05% as a cheap and safe drug to be used before MIGS insertion. Adrenaline decreases the risk of bleeding from the trabecular meshwork and also exhibits antifibrotic effects by arresting the cell cycle, thereby increasing the postoperative success rates in MIGS.

**Key message:**

***What is known***

- Fibrosis is the main cause of surgical failure in minimally invasive glaucoma surgery (MIGS). Mitomycin-C and 5-fluorouracil are too toxic to be used inside the eye.

***What is new***

- Adrenaline reduced the contractility of trabecular meshwork cells and inhibited the expression of key cell cycle genes and fibrosis genes, without significant cytotoxicity.
- Intracameral injection of adrenaline 0.05% did not result in any ocular or systemic adverse reactions in MIGS patients.

## Introduction

Glaucoma is the leading cause of irreversible blindness and currently affects 70 million people worldwide [1]. Nowadays, intraocular pressure (IOP) is no longer the only modifiable risk factor in the treatment of glaucoma. Numerous other risk factors can be treated, such as diabetes mellitus, arterial hypertension, and dyslipidemia, all of which have an unfavourable impact on primary open-angle glaucoma (POAG) neurodegeneration [2]. Conventional glaucoma filtration surgery, such as trabeculectomy and glaucoma drainage implant surgery, has been considered the gold standard in glaucoma surgical treatment [3]. Despite exhibiting efficacy at IOP reduction, these incisional surgeries are associated with potential postoperative blinding complications, such as hypotony, long-term risk of endophthalmitis and suprachoroidal haemorrhage [4]. In order to provide a less invasive and safer method for reducing IOP, newer surgical techniques, called minimally invasive glaucoma surgery (MIGS), have been developed and have found their place in the glaucoma treatment paradigm in the last two decades. MIGS refers to a group of IOP-lowering surgical interventions that enhance aqueous humour outflow through Schlemm's canal, the suprachoroidal space or the subconjunctival space [5]. Due to the small device geometry, which reduces trauma to ocular tissues and shortens operation time, MIGS offers enhanced safety profile, predictability, and minimal conjunctival manipulation [6]. MIGS can also be combined with phacoemulsification surgery in patients with both cataract and glaucoma.

There are multiple approaches to reduce IOP using MIGS devices. iStent® (Glaukos, Avedro) was the first approved ab interno MIGS implant for use in open-angle glaucoma [7]. It reduces IOP by increasing aqueous outflow from trabecular meshwork and Schlemm’s canal stents [8]. MINIject® (iSTAR Medical SA, Wavre, Belgium) is a new implant developed to target the suprachoroidal space [9]. Its unique flexible design conforms to the shape of the eye and the micropores promote aqueous outflow through the device [9]. Although the evidence supports a good safety profile of using MIGS devices in patients, fibrosis around the implants in the trabecular meshwork (TM) is the main cause of surgical failure in MIGS [10]. The significant postoperative fibrotic tissue formation due to cell proliferation and adhesion limits the success of newly created outflow routes, and makes several new micro-incision devices fail to receive market approval for clinical use [11, 12]. Currently, mitomycin-C (MMC) and 5-fluorouracil (5-FU) are commonly used as non-specific antifibrotic drugs after trabeculectomy. MMC inhibits fibroblast proliferation [13] and 5-FU interferes with cell growth [14]. However, they also carry the risk of severe tissue damage, corneal decompensation [15] and infections [16, 17], and are therefore too toxic to be used inside the eye in MIGS. Therefore, novel and non-toxic antifibrotic therapies are needed to enhance the long-term effectiveness of MIGS devices.

Adrenaline, also known as epinephrine, is endogenously produced by the adrenal glands [18] and has been shown to exhibit distinct antifibrotic effects in glaucoma surgery [19]. We recently investigated the antifibrotic effect of adrenaline by RNA-Sequencing technology and evaluated its impact on fibroblast contractility both in vitro and in three subconjunctival glaucoma surgeries (trabeculectomy, PreserFlo Microshunt, Baerveldt 350 tube surgery). Our results showed that adrenaline substantially decreased conjunctival fibroblast contractility without significant cytotoxicity even at high concentrations of 0.05%, and demonstrated that adrenaline may confer antifibrotic attributes in a concentration-dependent manner by affecting the expression of key cell cycle genes [20].

Given the promising effects in subconjunctival glaucoma surgery, the aim of this study was to investigate the potential benefits of adrenaline on Schlemm's canal and suprachoroidal MIGS devices. By probing the potential antifibrotic effects of adrenaline in these specific locations, this research endeavours to shed light on new strategies to increase the long-term success rates of Schlemm’s canal and suprachoroidal MIGS devices.

## Methods

### Cell culture

SV40-immortalized (NTM5) human TM cells were used in this study, and have been characterised and shown to be representative for TM cell studies [21, 22]. Technical replicates were also used in all assays. Human TM cells were cultured in an incubator at 37 °C with 5% CO_2_ and 95% humidity. The media for cell culture consisted of Dulbecco’s modified Eagle’s medium (DMEM) (Gibco, ThermoScientific, UK), 10% fetal calf serum (Gibco, ThermoScientific, UK), 100 units/mL penicillin and 0.1 mg/mL streptomycin (Sigma Aldrich, Gillingham, UK). All experiments were conducted in accordance with the Declaration of Helsinki and approved by the West of Scotland Research Ethics Committee (REC 19/WS/0146).

### Collagen contraction assay

A suspension containing 2 × 10^5^ TM cells was centrifuged at 1500 rpm for 5 min. The supernatant was discarded, and the cell pellet was resuspended in 100 µL of fetal calf serum. The collagen gel solution was prepared by 1 mL of Type 1 collagen (Fist Link, Wolverhampton, UK) and 160 µL of concentrated media, which consisted of 1.4 mL of DMEM 10 × (Sigma Aldrich, Gillingham, UK), 140 µL of L-glutamine (ThermoScientific, Loughborough, UK), and 360 µL of 7.5% sodium bicarbonate (Sigma Aldrich, Gillingham, UK). The pH was adjusted to 7.0 by sodium hydroxide before the cells were mixed with the collagen solution. 150 µL of the cell-gel mixture was placed in each MatTek dish and allowed to set for 10 min at 37 °C. The gels were treated with 1.5 mL of different concentrations of adrenaline (0%, 0.0005%, 0.01%) after carefully releasing the gels. The cells in the gels were observed using an Olympus CKX41 inverted microscope, and the gel photos were taken daily over 7 days and analysed using the ImageJ software. The percentage of matrix contraction was calculated using the formula:

$$Contraction\;(\%)=\left[\frac{Area\;of\;gel\;at\;Day\;0\;-\;Area\;of\;gel\;at\;Day\;n}{Area\;of\;gel\;at\;Day\;0}\right]\times 100$$

### Light microscopy observation

Human TM cells were seeded at a density of 1 × 10^5^ cells per well in 6-well plates and treated with different concentrations of adrenaline (0%, 0.0001%, 0.0005%, 0.001%, 0.005%, 0.01%). After 1-day treatment, cells were imaged using an Olympus CKX41 inverted microscope with Olympus CellSens Standard 1.13 software.

### Cell viability assay

Human TM cells were seeded at a density of 6.25 × 10^3^ cells per well in a 96-well plate and treated with different concentrations of adrenaline (0%, 0.0001%, 0.0005%, 0.001%, 0.005%, 0.01%) for 1 day. The drug solutions in the 96-well plate were then replaced by 100 µL of fresh media, followed by the addition of 20 µL of the Cell Titer 96 Aqueous one solution (Promega, Southampton, UK). The plate was incubated for 2 h at 37 °C and the absorbance was measured at 490 nm using a PHERAstar FS instrument (BMG Labtech, Aylesbury, UK). The cell viability was calculated as a percentage of the value of untreated (0%) cells.

### Real-time quantitative PCR

Human TM cells were seeded at a density of 1 × 10^5^ cells per well in 6-well plates and treated with different concentrations of adrenaline (0%, 0.0005%, 0.01%) for 1 day. The total RNA was extracted using a RNeasy mini kit (Qiagen, Crawley, UK) and the cDNA was synthesised using a high-capacity cDNA reverse transcription kit (ThermoScientific, Loughborough, UK). RT-qPCR was performed using the QuantiFast SYBR Green PCR kit (Qiagen, Crawley, UK) on a QuantStudio 7 Real-time PCR system. The reaction settings for 40 cycles were as follows: Holding stage: 50 °C for 2 min and 95 °C for 5 min; PCR stage: 95 °C for 5 min and 60 °C for 30 s. The sequences for the forward and reverse primers are presented in Table 1. The relative gene expression was calculated using the formula:

**Table 1:** Primer sequences for RT-qPCR

Gene names		Primer sequences
*ACTA2*	Forward	5’- AATGCAGAAGGAGATCACGC -3’
	Reverse	5’- TCCTGTTTGCTGATCCACATC -3’
*ASPM*	Forward	5'- TGCAGTGGGTGAACATGAAAA -3'
	Reverse	5'- CGAAGAGGGTGTTACCTCGTTT -3'
*AURKA*	Forward	5'- GGAGAGCTTAAAATTGCAGATTTTG -3'
	Reverse	5'- GCTCCAGAGATCCTTCTCAT -3'
*BRCA1*	Forward	5'- TTGTTACAAATCACCCCTCAAGG -3'
	Reverse	5'- CCCTGATACTTTTCTGGATGC -3'
*CCNB1*	Forward	5'- GTTATGCAGCACCTG -3'
	Reverse	5'- CTTGGCTAAATCTTGAACT -3'
*CDC20*	Forward	5’- GACCACTCCTAGCAAACCTGG -3'
	Reverse	5’- GGGCGTCTGGCTGTTTTCA -3'
*CDCA3*	Forward	5'- TGGTATTGCACGGACACCTA -3'
	Reverse	5'- TGTTTCACCAGTGGGCTTG -3'
*CDCA8*	Forward	5'- TTGACTACTTCGCCCTTG -3'
	Reverse	5'- CTTCTTCTTCCTCTTCCACTA -3'
*CEP55*	Forward	5'- TTTTTTTAAGTGAGATTTTATGTGG -3'
	Reverse	5'- CCATCCAATAAATAATACAAAACC -3'
*CLSPN*	Forward	5'- AGAGCAGCCACAATAGCAGC -3'
	Reverse	5'- ACGGCCTGTTTGTCTGTTGC -3'
*COL1A2*	Forward	5’- TGGATGAGGAGACTGGCAAC -3’
	Reverse	5’- TTAGAACCCCCTCCATCCCAC -3’
*DBF4*	Forward	5'- TAAGGATCTGGGAGGGCGAGT -3'
	Reverse	5'- ATGGCTGGGATGAGGTGAAGT -3'
*E2F7*	Forward	5'- GGAAAGGCAACAGCAAACTCT -3'
	Reverse	5'- TGGGAGAGCACCAAGAGTAGAAGA -3'
*E2F8*	Forward	5'- CCAACCCTGCTGTGAATA -3'
	Reverse	5'- TTTCTGGCTCATTACCCT -3'
*GAPDH*	Forward	5'- ACGGATTTGGTCGTATTGGGC -3'
	Reverse	5'- TTGACGGTGCCATGGAATTTG -3'
*KIF14*	Forward	5'- TGGTGAAATGGCCTGTACAAGT -3'
	Reverse	5'- GGCAACCAGTTAACCCTTTGAG -3'
*MKI67*	Forward	5'- CAGCACCTTTTCTCACCCTGG -3'
	Reverse	5'- AAACACGGGGGTAGCCCTTA -3'
*MRTFB*	Forward	5’- CTTCCTGTGGACTCCAGTG -3’
	Reverse	5’- TGTGACTCCTGACTCGCAG -3’
*PLK1*	Forward	5'- CGACTTCGTGTTCGTGGTG -3'
	Reverse	5'- AATAACTCGGTTTCGGTGC -3'
*PRR11*	Forward	5'- CGTATCTGCCACCGAGAACTT -3'
	Reverse	5'- GAGATGGTCTTCAGTGCTTCCT -3'

$$Relative\;Gene\;Expression={2}^{-(\Delta {Ct}_{target}-\Delta {Ct}_{GAPDH})}$$

### Intracameral injection of adrenaline during MIGS implantation

We tested the effects of adrenaline 0.05% intraoperatively in five patients with POAG undergoing a MIGS device combined with phacoemulsification. Two MIGS devices were included in this study: iStent inject® (Glaukos, Avedro) as a Schlemm’s canal MIGS and MINIject® (iSTAR Medical SA, Wavre, Belgium) as a suprachoroidal MIGS. Informed consent was obtained from all patients.

Phacoemulsification surgery was performed as per standard practice. Miochol was injected intracamerally at the end of phacoemulsification to constrict the pupil in preparation for MIGS implantation. All patients then received 0.1 mL of intracameral injection of adrenaline 0.05% before the anterior chamber was filled with viscoelastic and the MIGS device was inserted.

### Clinical examination

Clinical data, including best-corrected visual acuity (BCVA), IOP, central corneal thickness (CCT), cup-to-disc ratio (C/D), previous glaucoma surgery, previous glaucoma laser, lens status and anti-glaucoma medications were recorded preoperatively and postoperatively in all patients. Blood pressure, heart rate, and blood oxygen saturation were also measured preoperatively, intraoperatively, and postoperatively. Pupil diameter was measured using a measuring scale before and after surgery.

To record the positioning of the MIGS, the intraoperative insertion of the implant was captured by video recording during surgery. Postoperatively, gonioscopy was performed in clinic and photographs of the implant in the angle were taken by slit lamp camera (ARC slit lamp imaging camera, Carleton optical, Chesham, Buckinghamshire, UK). We also performed angle scans with an anterior segment OCT (ANTERION® Heidelberg Engineering, Germany) to visualise the implant transversally.

### Statistical analyses

GraphPad Prism was used for graph generation and statistical analysis. All graphs display mean and standard error of the mean (SEM). To determine statistical significance, a one-way ANOVA was performed and *P* values were calculated. *P* values, which were statistically significant, were expressed as follows: *P < 0.05, **P < 0.01, ***P < 0.001, ****P < 0.0001.

## Results

### Adrenaline significantly decreased matrix contraction in human TM cells

**Fig. 1:**
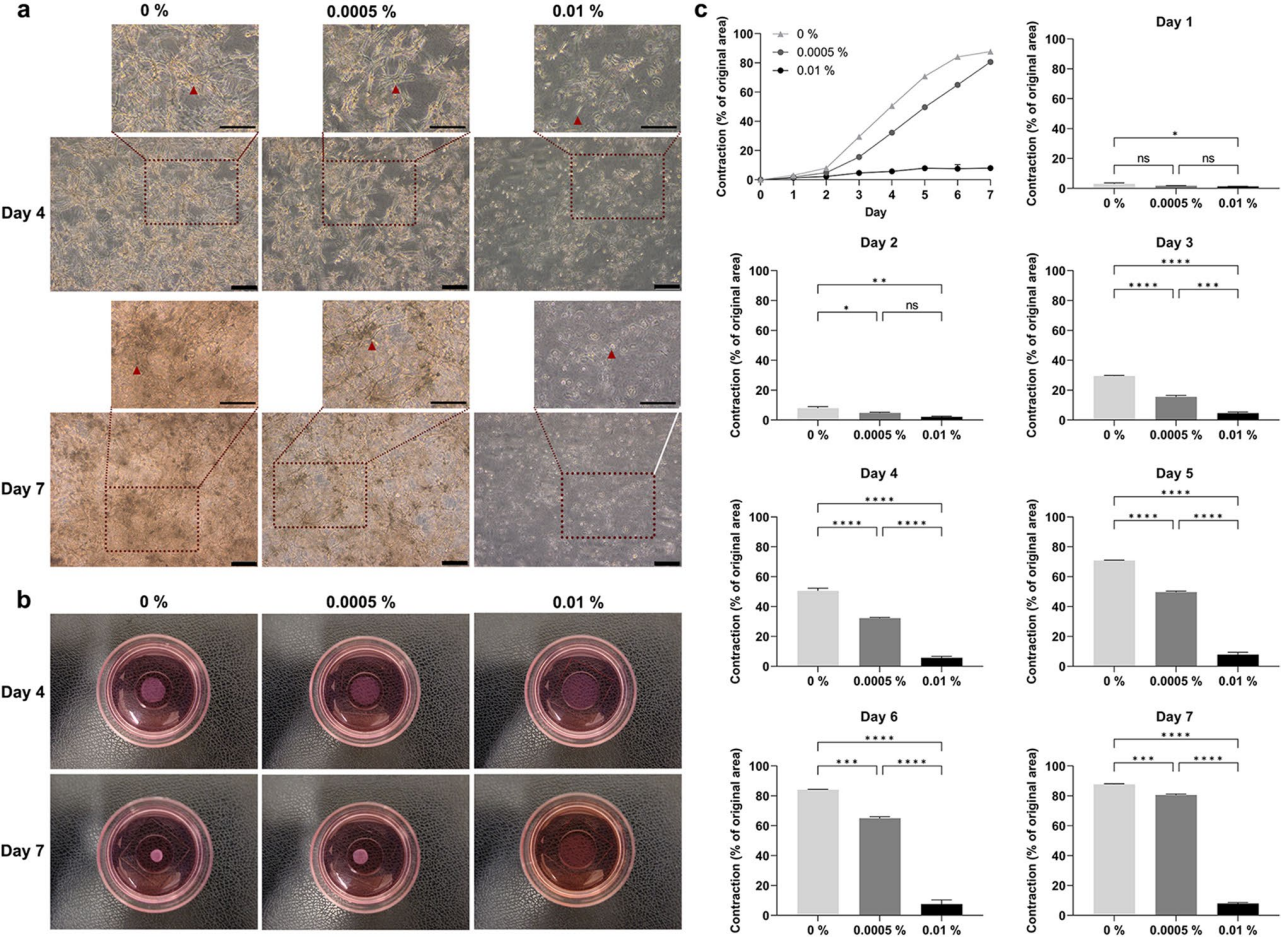
**a** Photomicrographs of human TM cells in collagen gels treated with adrenaline (0%, 0.0005%, 0.01%) at day 4 and day 7. Red arrows pointing at TM cells in the gels. Scale bar = 200 µm. **b** Representative gel photos at day 4 and day 7. **c** Three-dimensional collagen contraction assay of human TM cells treated with adrenaline (0%, 0.0005%, 0.01%) from day 1 to day 7. The percentage contraction was calculated from the original area on day 0. Results represent mean ± SEM for 3 independent replicates. ns, not significant; *ns* not significant, *P < 0.05, **P < 0.01, ***P < 0.001, ****P < 0.0001

A three-dimensional cell-populated collagen contraction assay was carried out to assess the contractility of TM cells after adrenaline treatment, as it has been shown to be a good in vitro model and functional assay to study tissue contraction in the eye [23, 24]. Increasing concentrations of adrenaline decreased the cell proliferation and cell density of TM cells in gels at both day 4 and day 7 (Fig. 1a). Figure 1b shows representative gel areas at day 4 and day 7. A dose-dependent decrease in collagen contraction was observed with increasing concentrations of adrenaline throughout the 7 days (Fig. 1c). At day 4, the TM cell-populated collagen gels treated with 0%, 0.0005%, and 0.01% adrenaline contracted 50.6%, 32.2%, and 5.7%, respectively, while at day 7, they contracted 87.8%, 80.6%, and 7.9%, respectively. The differences in collagen contraction were statistically significant for both 0.0005% adrenaline (*P* < 0.05 on day 2, *P* < 0.0001 on days 3–5, *P* < 0.001 on days 6–7) and 0.01% adrenaline (*P* < 0.05 on day 1, *P* < 0.01 on day 2, *P* < 0.0001 on days 3–7), when compared to no drug control 0%.

### Adrenaline did not affect cell viability and cell morphology in human TM cells

**Fig. 2:**
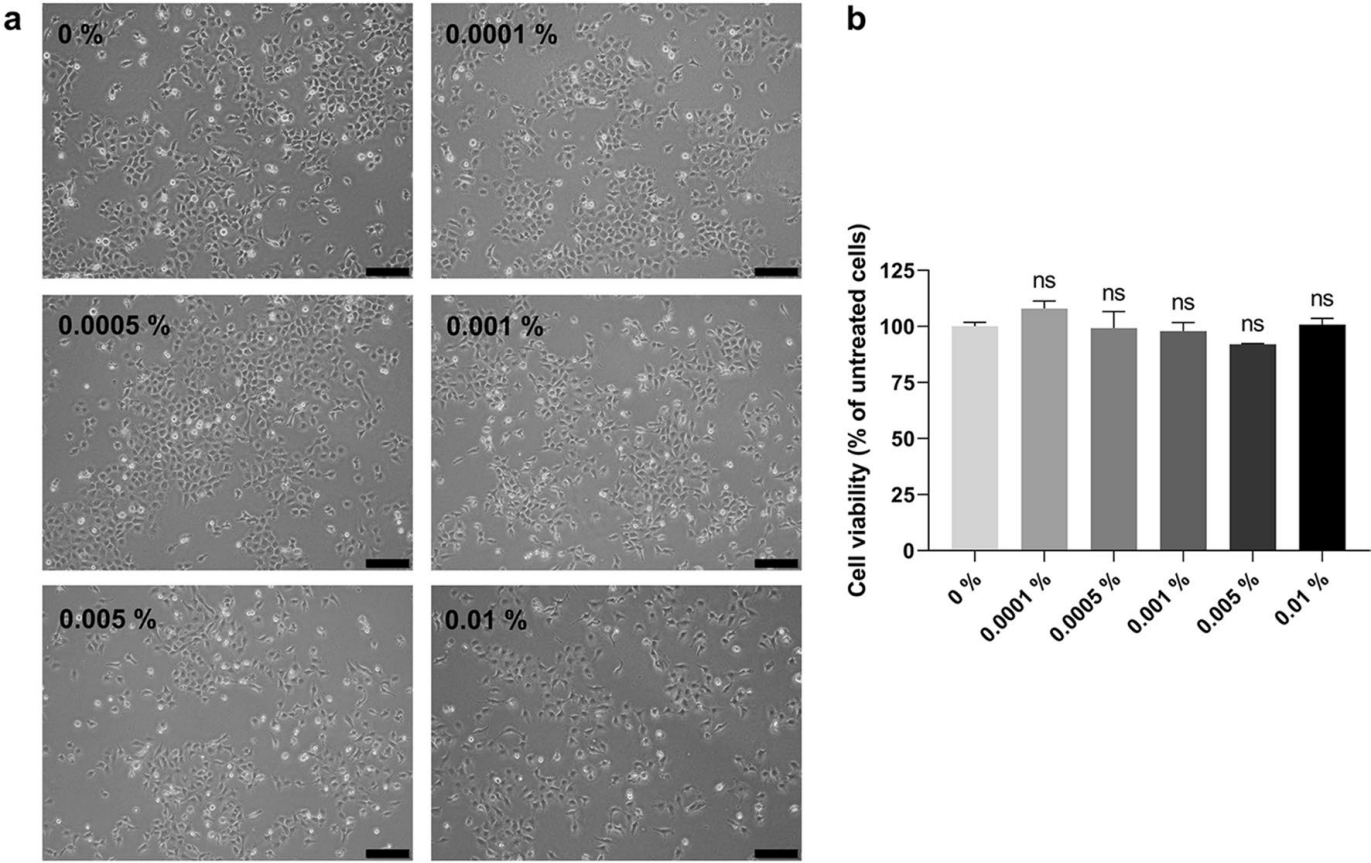
**a **Photomicrographs of human TM cells in 6-well plate after 1-day treatment with adrenaline (0%, 0.0001%, 0.0005%, 0.001%, 0.005%, 0.01%). Scale bar = 200 µm. **b** Cell viability of human TM cells after 1-day treatment with adrenaline (0%, 0.0001%, 0.0005%, 0.001%, 0.005%, 0.01%). Results represent mean ± SEM for 3 independent replicates. ns, not significant

The potential toxicity of adrenaline on TM cells was assessed by light microscopy observation and a cell viability assay. After 1-day treatment, the cell viability of adrenaline 0%, 0.0001%, 0.0005%, 0.001%, 0.005%, and 0.01% was 100.0%, 108.0%, 99.3%, 97.9%, 92.0%, and 100.7%, respectively. The TM cells on the 6-well plates did not display noticeable changes in cell morphology (Fig. 2a). All adrenaline concentrations (0.0001%, 0.0005%, 0.001%, 0.005%, 0.01%) did not exhibit a significant decrease in cell viability compared to no drug control 0% (*P* > 0.05) (Fig. 2b).

### Adrenaline significantly decreased the expression of key cell cycle genes in human TM cells

**Fig. 3:**
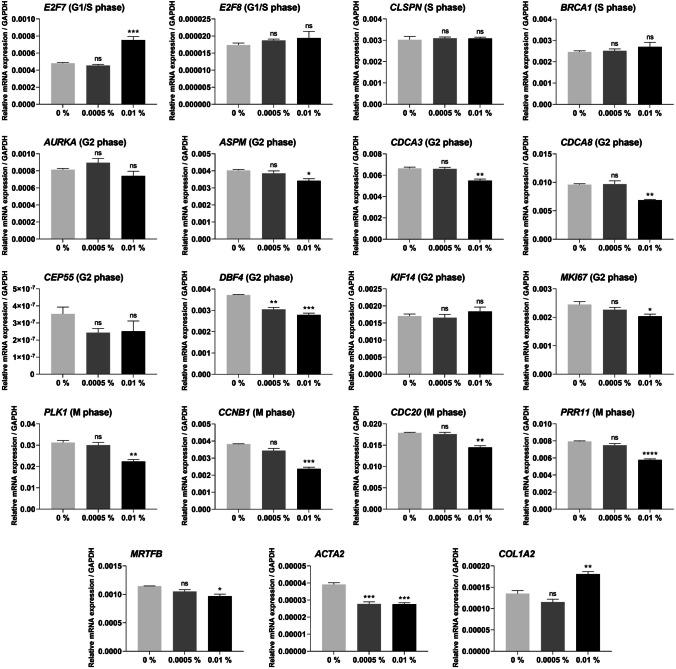
Gene expression in human TM cells after 1-day treatment with adrenaline (0%, 0.0005%, 0.01%). G1/S phase genes, including *E2F7* and *E2F8*. S phase genes, including *CLSPN* and *BRCA1*. G2 phase genes, including *AURKA*, *ASPM*, *CDCA3*, *CDCA8*, *CEP55*, *DBF4*, *KIF14*, and *MKI67*. M phase genes, including *PLK1*, *CCNB1*, *CDC20*, and *PRR11*. *MRTF-B*, *ACTA2*, and *COL1A2* genes. mRNA levels were normalised to *GAPDH*. Results represent mean ± SEM for 3 independent replicates. *ns* not significant, *P < 0.05, **P < 0.01, ***P < 0.001, ****P < 0.0001

We tested the effects of adrenaline on the expression of key cell cycle genes in human TM cells to investigate whether adrenaline would have similar effects as previously shown in human Tenon’s fibroblasts [20]. The expression of key cell cycle and fibrosis genes was measured by real-time qPCR after adrenaline treatment (Fig. 3). In the G2 phase, *ASPM* (*P* < 0.05), *CDCA3* (*P* < 0.01), *CDCA8* (*P* < 0.01), *DBF4* (*P* < 0.001), and *MKI67* (*P* < 0.05) genes were significantly downregulated after 0.01% adrenaline treatment when compared to no drug control 0%. The expression of *DBF4* gene also showed a significant decrease with 0.0005% adrenaline (*P* < 0.01).

In the M phase, *PLK1* (*P* < 0.01), *CCNB1* (*P* < 0.001), *CDC20* (*P* < 0.01), and *PRR11* (*P* < 0.0001) genes were significantly downregulated after 0.01% adrenaline treatment when compared to no drug control 0%, but no significant differences were observed in the 0.0005% adrenaline group. In addition, *MRTFB* showed a significantly reduced gene expression with 0.01% adrenaline (*P* < 0.05), and *ACTA2* showed a decreased gene expression with both 0.01% and 0.0005% adrenaline (*P* < 0.001).

### Intracameral adrenaline injection did not cause any ocular adverse effects during and after MIGS implantation

We next validated our results by injecting adrenaline 0.05% intracamerally in patients with POAG receiving a Schlemm’s canal MIGS (iStent inject) or suprachoroidal MIGS (MINIject), combined with phacoemulsification surgery. Demographically, we gathered data from different age and ethnic groups, which were evenly distributed between both MIGS groups (Table 2). All patients in the study were using multiple topical antiglaucoma medications, but none were on oral acetazolamide tablets.

**Table 2:** Patient clinical details

Clinical data	MINIject	iStent inject
Patients	1, 2, 3	4, 5
Type of glaucoma	POAG	POAG
Gender	Female	Female
Mean age in years	58	61
Ethnicity	1 White-Caucasian, 1 Afro-Caribbean and 1 Asian	2 Afro-Caribbean
Mean BCVA (LogMAR) ± SEM	0.2 ± 0.2	0.4 ± 0.2
Mean IOP (mmHg) ± SEM	15.0 ± 1.0	21.5 ± 5.5
Mean CCT (µm) ± SEM	530.0 ± 32.6	564.5 ± 18.5
Mean Cup to Disc ratio ± SEM	0.73 ± 0.03	0.85 ± 0.05
Previous glaucoma surgery	Nil	Nil
Previous glaucoma laser	Nil	SLT (patient 5)
Mean number of anti-glaucoma drops ± SEM	3.0 ± 0.1	3.0 ± 0.1
Prostaglandin analogue drop, mean duration	Yes, 6 years 3 months	Yes, 5 years 5 months
Beta blocker drop, mean duration	Yes, 7 years 1 month	Yes, 6 years 4 months
Carbonic anhydrase inhibitor drop, mean duration	Yes, 5 years 3 months	Yes, 6 years 11 months
Alpha agonist drop, mean duration	Yes, 2 years 2 months	No
Acetazolamide tablet, mean duration	No	No

*POAG* primary open-angle glaucoma, *BCVA* best corrected visual acuity, *LogMAR* logarithm of the minimum angle of resolution, *IOP* intraocular pressure, *CCT* central corneal thickness

**Fig. 4:**
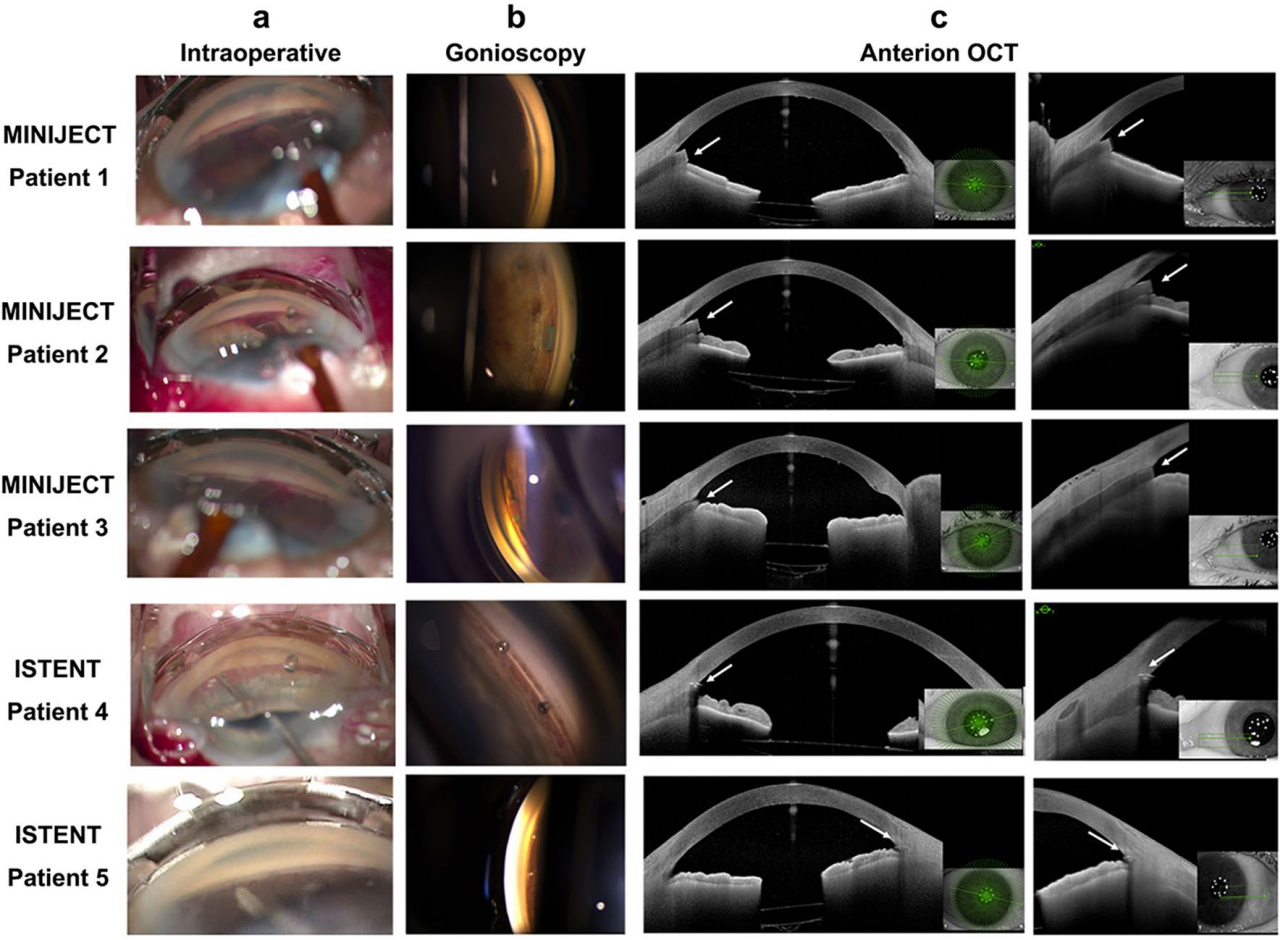
Injection of intracameral adrenaline prior to MIGS implantation. Patients 1, 2 and 3 received MINIject implantation. Patients 4 and 5 received iStent inject implantation. **a** Intraoperative view of MIGS implantation using a gonio lens. **b** Gonioscopic view of the MIGS in clinic at 1 week postoperatively. **c** Anterior segment OCT of the angle after MIGS implantation using Anterion OCT at 1 week postoperatively. White arrows show positioning of the iStent inject or MINIject

Adrenaline exhibited vasoconstrictive effects and significantly reduced intraoperative bleeding in both iStent inject and MINIject patients (Fig. 4a). None of the patients experienced any intraoperative or postoperative adverse effects due to the intracameral adrenaline 0.05% injection. The MINIject patients (patients 1, 2, 3) had preoperative BCVA (logMAR) of 0.0, 0.6 and 0.1, respectively. For these patients, preoperative IOP was 17, 14 and 14 mmHg, respectively, on at least two anti-glaucoma drops. CCT was found to be 513, 484 and 593 µm at baseline for these patients, and their C/D ratio was 0.8, 0.7 and 0.7, respectively (Table 2). One week postoperatively, BCVA was 0.2, 0.6 and 0.0 for patients 1, 2 and 3, respectively. Postoperative IOP was 9, 15 and 16 mmHg on no antiglaucoma medications. The implants were well positioned on gonioscopy (Fig. 4b) and the surrounding trabecular meshwork tissues were healthy (Fig. 4c).

The iStent inject patients (patients 4 and 5) had preoperative BCVA (logMAR) of 0.5 and 0.2, respectively. For these patients, preoperative IOP was 16 and 27 mmHg, respectively, on at least two anti-glaucoma drops. Their CCT was 546 and 583 µm at baseline, and their C/D ratio was 0.9 and 0.8, respectively (Table 2). One week postoperatively, BCVA was 0.5 and 0.2, and IOP was 16 and 14 mmHg, respectively, on no antiglaucoma medications. The implants were correctly positioned (Fig. 4b) and the surrounding trabecular meshwork tissues were also healthy (Fig. 4c).

### Intracameral adrenaline injection did not significantly affect blood pressure, heart rate, oxygen saturation and pupil size during MIGS implantation

All patients had stable blood pressures, heart rates, and oxygen saturations preoperatively, during surgery and postoperatively. Before surgery, the MINIject patients (patients 1, 2, 3) showed a blood pressure of 142/85, 124/84 and 176/80 mmHg, respectively. Their preoperative heart rate was 73, 90 and 72 beats per minute, and their oxygen saturation was 98%, 96% and 99%, respectively. The iStent inject patients (patients 4 and 5) had a preoperative blood pressure of 185/105 and 179/94 mmHg. Their heart rate was 93 and 77 beats per minute, and their oxygen saturation was 96% and 99%, respectively.

During surgery, the MINIject patients (patients 1, 2, 3) showed a blood pressure of 143/74, 154/71 and 176/76 mmHg, respectively. Intraoperatively, their heart rate was 60, 93 and 70 beats per minute, and their oxygen saturation was 100%, 97% and 97%, respectively. For the patients who received iStent inject (patients 4 and 5), the blood pressure was 185/105 and 179/94 mmHg during surgery. Intraoperatively, their heart rate was 84 and 59 beats per minute, and their oxygen saturation was 96% and 99%, respectively.

After surgery, the MINIject patients (patients 1, 2, 3) showed a blood pressure of 140/73, 154/88 and 176/76 mmHg, respectively. Postoperatively, their heart rate was 60, 93 and 66 beats per minute for the same patients, respectively. Their oxygen saturation after surgery was 98%, 97% and 96%, respectively. For patients who received iStent inject (patients 4 and 5), the blood pressure was 185/100 and 179/94 mmHg after surgery. Postoperatively, their heart rate was 95 and 89 beats per minute, and their oxygen saturation was 96% and 95%, respectively.

All patients received intracameral miochol as standard preoperative drug before MIGS implantation. There was no significant change in pupil diameter observed intraoperatively and postoperatively after intracameral adrenaline 0.05% injection, remaining between 6 and 7 mm in diameter in all patients.

## Discussion

Fibrosis is the most important cause of failure after MIGS. Although the antimetabolites MMC and 5-FU are commonly used to modulate wound healing after subconjunctival glaucoma filtration surgery, they are too toxic to be used intraocularly in MIGS due to the severe sight-threatening side effects, such as hypotonous maculopathy [25], corneal melting and perforation [26], and scleral calcification [27]. Therefore, there is a large unmet clinical need to develop an alternative and non-toxic antifibrotic drug that can be used in MIGS.

Adrenaline is a safe, cost-effective, and widely available drug in ophthalmic surgery, and has been shown to have a beneficial antifibrotic effect in human Tenon’s fibroblasts [20]. Severe fibrosis of the TM tissue can lead to sustained extracellular matrix accumulation and distortion of the TM framework, resulting in increased resistance to aqueous humour outflow and elevated IOP [10]. It also has an adverse impact on the implantation and is the primary cause of MIGS failure [22]. Our in vitro results indicate that adrenaline also exhibits concentration-dependent antifibrotic effects in human TM cells. Adrenaline 0.0005% and 0.01% significantly decreased the contractility of TM cells by 7.2% and 79.8%, respectively, after a 7-day contraction assay. Meanwhile, no apparent cytotoxicity was observed in TM cells after 1-day treatment with different concentrations of adrenaline (0.0001%, 0.0005%, 0.001%, 0.005%, 0.01%). These findings highlight the potential application of adrenaline in MIGS for its antifibrotic and vasoconstrictor effects, thereby enhancing the surgical success rates of MIGS.

Our previous RNA-Sequencing results provide compelling evidence that high concentrations of adrenaline have an impact on cell cycle genes in human Tenon’s fibroblasts [20, 28, 29]. We further examined the expression level of key cell cycle genes and fibrosis-related genes in TM cells after adrenaline treatment. In the G2 phase, *ASPM*, *CDCA3*, *CDCA8*, *DBF4*, and *MKI67* genes were significantly downregulated by adrenaline 0.01%. *ASPM* is involved in the formation of the mitotic spindle [30]. *CDCA3* is a crucial regulator of cell cycle progression from the G2 phase to mitosis [31]. *CDCA8* is also a component of the chromosomal passenger complex and is important in mitosis [32]. *DBF4* plays a vital role in DNA replication [33], while *MKI67* is closely related to cell proliferation [34]. In the M phase, adrenaline 0.01% significantly decreased the gene expression of *PLK1*, *CCNB1*, *CDC20*, *PRR11*. *PLK1* is a significant target of the DNA damage checkpoint, allowing cell-cycle arrests at multiple points in the G2 phase and mitosis [35]. *CCNB1* is essential for the proper control of the G2/M transition phase[36]. *CDC20* also plays a vital role in both nuclear movement prior to anaphase and chromosome separation [37]. *PRR11* plays a pivotal role in the accurate regulation from the late S phase to mitosis [38]. The downregulation of these genes indicates that adrenaline inhibits cell cycle progression and suppresses the proliferation of TM cells, causing them to enter a growth arrest phase without undergoing cell death.

Moreover, adrenaline 0.01% significantly reduced the expression of *MRTFB* and *ACTA2* genes, while *ACTA2* also exhibited decreased level after treatment with adrenaline 0.0005%. *MRTFB* is one of the major regulators of cytoskeletal gene expressions and is essential for TGF-β-induced fibrosis [39]. *ACTA2* is an important downstream gene of the *MRTFB/SRF* pathway and encodes α-SMA, which plays an important role in myofibroblast differentiation [40, 41]. In this study, the lower expression of *MRTFB* and *ACTA2* genes after adrenaline treatment was consistent with the decreased matrix contraction, indicating a decrease in fibrosis development in TM cells.

To validate the feasibility of adrenaline application in MIGS, we further examined the safety and efficacy of adrenaline in POAG patients undergoing Schlemm’s canal MIGS (iStent inject) or suprachoroidal MIGS (MINIject), when combined with phacoemulsification surgery. Different concentrations of adrenaline have been applied in the clinical setting. Adrenaline can be injected into the anterior chamber [42] or added in the infusion solution during cataract surgery [43], in order to achieve adequate and sustained pupillary dilation. When used in combination with atropine, adrenaline is also effective in the management of intraoperative floppy-iris syndrome due to a powerful synergistic effect on iris dilation[44]. Adrenaline 0.1% exhibits a good safety profile with no impact on blood pressure and heart rate [42, 45–47], and adrenaline 0.02% does not increase the risk of postoperative macular oedema [48]. Based on these previous studies, we selected an adrenaline concentration of 0.05% for intracameral injection (0.1 mL) in patients just prior to MIGS implantation in this study.

None of the patients experienced any intraoperative or postoperative adverse reactions. Patients receiving these two MIGS devices all exhibited stable blood pressures, heart rates, and oxygen saturations during and after surgery, demonstrating a good safety profile of intracameral adrenaline 0.05%. Patient 2 showed a 30 mmHg increase in blood pressure from preoperative stage to intraoperative stage, which was related to the patient being very anxious. Although adrenaline may induce adverse cardiovascular effects, its systemic absorption is limited [38], likely due to the presence of the blood-retinal barrier. Nonetheless, it remains contraindicated in patients with hypertension, heart disease, arrhythmias, and narrow angle glaucoma, and the use of adrenaline in these patients requires thorough patient assessment and careful patient selection [20].

The study had a few limitations. While transformed TM cells were used in this study, they may differ from primary TM cells in specific responses. Thus, it would be valuable to include primary TM cells in future in vitro studies. Although the data were carefully collected and analysed, this study was a proof-of-concept study and the sample size of patients was relatively small. With a known genetic variation in the alpha1B-adrenergic receptor [49], ethnic differences in adrenaline sensitivity also require further consideration. Additionally, the findings presented are specific to POAG patients and two MIGS devices (iStent inject and MINIject), which may not be directly transferable to other MIGS devices. Including individuals of different ethnicities and patients with other types of glaucoma, as well as incorporating a broader range of MIGS devices available on the market, could provide a more comprehensive evaluation for the use of adrenaline in MIGS in the future. Furthermore, this is a short-term study and the early incidence of stent failure is lower than the later failure rate. Currently, it is unknown whether adrenaline affects the cell cycle of other eye cells, such as potentially blocking any stem cell repopulation effects. Further research is needed to determine if this effect could lead to adverse consequences. It also remains unclear whether adrenaline is associated with a better reduction in IOP over time, which requires longer prospective studies. This study is focused on the early fibrotic responses, and moving beyond short-term effectiveness will be crucial to examine longer-term impacts. Given that endothelin-1 (ET-1) is a vasoconstrictor produced by endothelial cells, and that adrenaline can enhance its secretion, measuring ET-1 levels in the aqueous humour might help further understand the mechanism of adrenaline’s vasoconstrictive effects and its impact on aqueous humour outflow in the future.

In conclusion, this study showed that adrenaline reduced the contractility of TM cells in a dose-dependent manner, and suppressed the expression of key cell cycle and fibrosis genes with no significant cytotoxicity. The intracameral injection of adrenaline 0.05% in patients undergoing MIGS implantation also demonstrated a good safety profile during and after surgery. Unless contraindicated, we therefore recommend intracameral injections of adrenaline 0.05% as a cheap and safe drug to be used just before MIGS insertion, as it decreases the risk of bleeding from the trabecular meshwork and also exhibits antifibrotic effects by arresting the cell cycle, thereby increasing the postoperative success rates in MIGS.

## References

[1] Weinreb RN, Aung T, Medeiros FA (2014) The pathophysiology and treatment of glaucoma: a review. JAMA 311:1901–1911. 10.1001/jama.2014.3192 24825645 10.1001/jama.2014.3192 PMC4523637

[2] Michels TC, Ivan O (2023) Glaucoma: Diagnosis and Management. Am Fam Physician 107:253–262 36920817

[3] Lim R (2022) The surgical management of glaucoma: A review. Clin Exp Ophthalmol 50:213–231. 10.1111/ceo.14028 35037376 10.1111/ceo.14028

[4] Larsen CL, Samuelson TW (2017) Managing coexistent cataract and glaucoma with iStent. Surv Ophthalmol 62:706–711. 10.1016/j.survophthal.2016.01.006 27217122 10.1016/j.survophthal.2016.01.006

[5] Lee RMH, Bouremel Y, Eames I, Brocchini S, Khaw PT (2020) Translating minimally invasive glaucoma surgery devices. Clin Transl Sci 13:14–25. 10.1111/cts.12660 31568666 10.1111/cts.12660 PMC6951459

[6] Schehlein EM, Kaleem MA, Swamy R, Saeedi OJ (2017) Microinvasive glaucoma surgery: an evidence-based assessment. Expert Rev Ophthalmol 12:331–343. 10.1080/17469899.2017.1335597 30026790 10.1080/17469899.2017.1335597 PMC6049090

[7] Shalaby WS, Jia J, Katz LJ, Lee D (2021) iStent inject: comprehensive review. J Cataract Refract Surg 47:385–399. 10.1097/j.jcrs.0000000000000325 32842078 10.1097/j.jcrs.0000000000000325

[8] Gurnani B, Tripathy K (2024) Minimally invasive glaucoma surgery. In: StatPearls [Internet]. Treasure Island (FL): StatPearls Publishing. Available from: https://www.ncbi.nlm.nih.gov/books/NBK582156/35881761

[9] Denis P, Hirneiß C, Durr GM, Reddy KP, Kamarthy A, Calvo E, Hussain Z, Ahmed IK (2022) Two-year outcomes of the MINIject drainage system for uncontrolled glaucoma from the STAR-I first-in-human trial. Br J Ophthalmol 106:65–70. 10.1136/bjophthalmol-2020-316888 33011690 10.1136/bjophthalmol-2020-316888 PMC8685654

[10] Qin M, Yu-Wai-Man C (2023) Glaucoma: Novel antifibrotic therapeutics for the trabecular meshwork. Eur J Pharmacol 954:175882. 10.1016/j.ejphar.2023.175882 37391006 10.1016/j.ejphar.2023.175882 PMC10804937

[11] Rekas M, Pawlik B, Grala B, Kozlowski W (2013) Clinical and morphological evaluation of gold micro shunt after unsuccessful surgical treatment of patients with primary open-angle glaucoma. Eye (Lond) 27:1214–1217. 10.1038/eye.2013.154 23867717 10.1038/eye.2013.154 PMC3806560

[12] Schmidt W, Kastner C, Sternberg K, Allemann R, Löbler M, Guthoff R, Schmitz KP (2013) New concepts for glaucoma implants–controlled aqueous humor drainage, encapsulation prevention and local drug delivery. Curr Pharm Biotechnol 14:98–111 23092262

[13] Wang YW, Ren JH, Xia K, Wang SH, Yin TF, Xie DH, Li LH (2012) Effect of mitomycin on normal dermal fibroblast and HaCat cell: an in vitro study. J Zhejiang Univ Sci B 13:997–1005. 10.1631/jzus.B1200055 23225855 10.1631/jzus.B1200055 PMC3520454

[14] Ghiringhelli F, Apetoh L (2015) Enhancing the anticancer effects of 5-fluorouracil: current challenges and future perspectives. Biomed J 38:111–116. 10.4103/2319-4170.130923 25163503 10.4103/2319-4170.130923

[15] Mohammadpour M, Jabbarvand M, Javadi MA (2007) Focal corneal decompensation after filtering surgery with mitomycin C. Cornea 26:1285–1287. 10.1097/ICO.0b013e318150d371 18043196 10.1097/ICO.0b013e318150d371

[16] Mearza AA, Aslanides IM (2007) Uses and complications of mitomycin C in ophthalmology. Expert Opin Drug Saf 6:27–32. 10.1517/14740338.6.1.27 17181449 10.1517/14740338.6.1.27

[17] Franks WA, Hitchings RA (1991) Complications of 5–fluorouracil after trabeculectomy. Eye (Lond) 5(Pt 4):385–389. 10.1038/eye.1991.63 1743353 10.1038/eye.1991.63

[18] Dalal R, Grujic D (2024) Epinephrine. In: StatPearls [Internet]. Treasure Island (FL): StatPearls Publishing. Available from: https://www.ncbi.nlm.nih.gov/books/NBK482160/29489283

[19] Young TL, Higginbotham EJ, Zou XL, Farber MD (1990) Effects of topical glaucoma drugs on fistulized rabbit conjunctiva. Ophthalmology 97:1423–1427. 10.1016/s0161-6420(90)32392-8 2255514 10.1016/s0161-6420(90)32392-8

[20] Thong KX, Andriesei P, Luo J, Qin M, Ng J, Tagalakis AD, Hysi P, Yu-Wai-Man C (2023) Adrenaline blocks key cell cycle genes and exhibits antifibrotic and vasoconstrictor effects in glaucoma surgery. Exp Eye Res 233:109561. 10.1016/j.exer.2023.109561 37429521 10.1016/j.exer.2023.109561

[21] Kennedy SM, Sheridan C, Kearns VR, Bilir EK, Fan X, Grierson I, Choudhary A (2019) Thrombospondin-2 is up-regulated by TGFβ2 and increases fibronectin expression in human trabecular meshwork cells. Exp Eye Res 189:107820. 10.1016/j.exer.2019.107820 31589839 10.1016/j.exer.2019.107820

[22] Luo J, Tan G, Thong KX, Kafetzis KN, Vallabh N, Sheridan CM, Sato Y, Harashima H, Tagalakis AD, Yu-Wai-Man C (2022) Non-Viral Gene Therapy in Trabecular Meshwork Cells to Prevent Fibrosis in Minimally Invasive Glaucoma Surgery. Pharmaceutics 14 10.3390/pharmaceutics14112472 10.3390/pharmaceutics14112472 PMC9693853 36432663

[23] Dahlmann-Noor AH, Martin-Martin B, Eastwood M, Khaw PT, Bailly M (2007) Dynamic protrusive cell behaviour generates force and drives early matrix contraction by fibroblasts. Exp Cell Res 313:4158–4169. 10.1016/j.yexcr.2007.07.040 17869245 10.1016/j.yexcr.2007.07.040 PMC2764386

[24] Daniels JT, Cambrey AD, Occleston NL, Garrett Q, Tarnuzzer RW, Schultz GS, Khaw PT (2003) Matrix metalloproteinase inhibition modulates fibroblast-mediated matrix contraction and collagen production in vitro. Invest Ophthalmol Vis Sci 44:1104–1110. 10.1167/iovs.02-0412 12601036 10.1167/iovs.02-0412

[25] Singh J, O'Brien C, Chawla HB (1995) Success rate and complications of intraoperative 0.2 mg/ml mitomycin C in trabeculectomy surgery. Eye (Lond) 9 ( Pt 4): 460–466 10.1038/eye.1995.107 10.1038/eye.1995.107 7498567

[26] Dougherty PJ, Hardten DR, Lindstrom RL (1996) Corneoscleral melt after pterygium surgery using a single intraoperative application of mitomycin-C. Cornea 15:537–540 8862932

[27] Rubinfeld RS, Pfister RR, Stein RM, Foster CS, Martin NF, Stoleru S, Talley AR, Speaker MG (1992) Serious complications of topical mitomycin-C after pterygium surgery. Ophthalmology 99:1647–1654. 10.1016/s0161-6420(92)31749-x 1454338 10.1016/s0161-6420(92)31749-x

[28] Bar-Joseph Z, Siegfried Z, Brandeis M, Brors B, Lu Y, Eils R, Dynlacht BD, Simon I (2008) Genome-wide transcriptional analysis of the human cell cycle identifies genes differentially regulated in normal and cancer cells. Proc Natl Acad Sci U S A 105:955–960. 10.1073/pnas.0704723105 18195366 10.1073/pnas.0704723105 PMC2242708

[29] Giotti B, Joshi A, Freeman TC (2017) Meta-analysis reveals conserved cell cycle transcriptional network across multiple human cell types. BMC Genomics 18:30. 10.1186/s12864-016-3435-2 28056781 10.1186/s12864-016-3435-2 PMC5217208

[30] Zhong X, Liu L, Zhao A, Pfeifer GP, Xu X (2005) The abnormal spindle-like, microcephaly-associated (ASPM) gene encodes a centrosomal protein. Cell Cycle 4:1227–1229. 10.4161/cc.4.9.2029 16123590 10.4161/cc.4.9.2029

[31] Ayad NG, Rankin S, Murakami M, Jebanathirajah J, Gygi S, Kirschner MW (2003) Tome-1, a trigger of mitotic entry, is degraded during G1 via the APC. Cell 113:101–113. 10.1016/s0092-8674(03)00232-0 12679038 10.1016/s0092-8674(03)00232-0

[32] Wu Y, Zeng S, Miao C, Wu H, Xu X, Chen L, Lu G, Lin G, Dai C (2023) A 1-kb human CDCA8 promoter directs the spermatogonia-specific luciferase expression in adult testis. Gene 866:147350. 10.1016/j.gene.2023.147350 36898512 10.1016/j.gene.2023.147350

[33] Li N, Gao N, Zhai Y (2023) DDK promotes DNA replication initiation: Mechanistic and structural insights. Curr Opin Struct Biol 78:102504. 10.1016/j.sbi.2022.102504 36525878 10.1016/j.sbi.2022.102504

[34] Du Y, Cai Y, Lv Y, Zhang L, Yang H, Liu Q, Hong M, Teng Y, Tang W, Ma R, Wu J, Wu J, Wang Q, Chen H, Li K, Feng J (2022) Single-cell RNA sequencing unveils the communications between malignant T and myeloid cells contributing to tumor growth and immunosuppression in cutaneous T-cell lymphoma. Cancer Lett 551:215972. 10.1016/j.canlet.2022.215972 36265653 10.1016/j.canlet.2022.215972

[35] Smits VA, Klompmaker R, Arnaud L, Rijksen G, Nigg EA, Medema RH (2000) Polo-like kinase-1 is a target of the DNA damage checkpoint. Nat Cell Biol 2:672–676. 10.1038/35023629 10980711 10.1038/35023629

[36] Hwang A, McKenna WG, Muschel RJ (1998) Cell cycle-dependent usage of transcriptional start sites. A novel mechanism for regulation of cyclin B1. J Biol Chem 273:31505–31509. 10.1074/jbc.273.47.31505 9813064 10.1074/jbc.273.47.31505

[37] Yu H (2007) Cdc20: a WD40 activator for a cell cycle degradation machine. Mol Cell 27:3–16. 10.1016/j.molcel.2007.06.009 17612486 10.1016/j.molcel.2007.06.009

[38] Zhang C, Zhang Y, Li Y, Zhu H, Wang Y, Cai W, Zhu J, Ozaki T, Bu Y (2015) PRR11 regulates late-S to G2/M phase progression and induces premature chromatin condensation (PCC). Biochem Biophys Res Commun 458:501–508. 10.1016/j.bbrc.2015.01.139 25666944 10.1016/j.bbrc.2015.01.139

[39] Wang DZ, Li S, Hockemeyer D, Sutherland L, Wang Z, Schratt G, Richardson JA, Nordheim A, Olson EN (2002) Potentiation of serum response factor activity by a family of myocardin-related transcription factors. Proc Natl Acad Sci U S A 99:14855–14860. 10.1073/pnas.222561499 12397177 10.1073/pnas.222561499 PMC137508

[40] Chen M, Liu J, Yang W, Ling W (2017) Lipopolysaccharide mediates hepatic stellate cell activation by regulating autophagy and retinoic acid signaling. Autophagy 13:1813–1827. 10.1080/15548627.2017.1356550 29160747 10.1080/15548627.2017.1356550 PMC5788469

[41] Hetzler PT 3rd, Dash BC, Guo S, Hsia HC (2019) Targeting Fibrotic Signaling: A Review of Current Literature and Identification of Future Therapeutic Targets to Improve Wound Healing. Ann Plast Surg 83:e92–e95. 10.1097/sap.0000000000001955 31246672 10.1097/SAP.0000000000001955 PMC6851445

[42] Suresha AR, Gunashree KN (2021) Safety and efficacy of intracameral mydriatics: Lignocaine and epinephrine in manual small-incision cataract surgery and their effect on blood pressure and heart rate. Indian J Ophthalmol 69:1343–1345. 10.4103/ijo.IJO_3428_20 33913904 10.4103/ijo.IJO_3428_20 PMC8186606

[43] Alabdulwahhab KM (2021) Efficacy and effect of intracameral adrenaline infusion on pulse rate and blood pressure during phacoemulsification in patients with dark irides. Eur Rev Med Pharmacol Sci 25:4773–4778. 10.26355/eurrev_202101_26389 34337725 10.26355/eurrev_202101_26389

[44] Masket S, Belani S (2007) Combined preoperative topical atropine sulfate 1% and intracameral nonpreserved epinephrine hydrochloride 1:4000 [corrected] for management of intraoperative floppy-iris syndrome. J Cataract Refract Surg 33:580–582. 10.1016/j.jcrs.2006.10.059 17397728 10.1016/j.jcrs.2006.10.059

[45] Shugar JK (2006) Use of epinephrine for IFIS prophylaxis. J Cataract Refract Surg 32:1074–1075. 10.1016/j.jcrs.2006.01.110 16857480 10.1016/j.jcrs.2006.01.110

[46] Cionni RJ, Barros MG, Kaufman AH, Osher RH (2003) Cataract surgery without preoperative eyedrops. J Cataract Refract Surg 29:2281–2283. 10.1016/j.jcrs.2003.09.009 14709286 10.1016/j.jcrs.2003.09.009

[47] Visco D (2018) Effect of phenylephrine/ketorolac on iris fixation ring use and surgical times in patients at risk of intraoperative miosis. Clin Ophthalmol 12:301–305. 10.2147/OPTH.S149522 29440873 10.2147/OPTH.S149522 PMC5804732

[48] Bozkurt E, Yazici AT, Pekel G, Albayrak S, Cakir M, Pekel E, Yilmaz OF (2010) Effect of intracameral epinephrine use on macular thickness after uneventful phacoemulsification. J Cataract Refract Surg 36:1380–1384. 10.1016/j.jcrs.2010.02.020 20656163 10.1016/j.jcrs.2010.02.020

[49] Adefurin A, Ghimire LV, Kohli U, Muszkat M, Sofowora GG, Li C, Levinson RT, Paranjape SY, Stein CM, Kurnik D (2017) Genetic variation in the alpha(1B)-adrenergic receptor and vascular response. Pharmacogenomics J 17:366–371. 10.1038/tpj.2016.29 27089938 10.1038/tpj.2016.29 PMC5071105

